# The gut microbiome of obese postpartum women with and without previous gestational diabetes mellitus and the gut microbiota of their babies

**DOI:** 10.1186/s13098-022-00954-2

**Published:** 2022-12-24

**Authors:** Patricia Medici Dualib, Gabriel Fernandes, Carla R. Taddei, Camila R. S. Carvalho, Luiz Gustavo Sparvoli, Célia Bittencourt, Isis T. Silva, Rosiane Mattar, Sandra R. G. Ferreira, Sergio A. Dib, Bianca de Almeida-Pititto

**Affiliations:** 1 Department of Medicine, Escola Paulista Medicina, Universidade Federal de São Paulo, Rua Sena Madureira, 1500, Vila Clementino, São Paulo, SP CEP 04021-001 Brazil; 2 DepaBiosystems Informatics and Genomics Group, Instituto René Rachou – Fiocruz Minas, Av Augusto de Lima, 1714, Belo Horizonte, MG CEP 30190-002 Brazil; 3 Department of Clinical and Toxicological Analysis, Universidade de São Paulo (USP), Av. Prof. Lineu Prestes 580-Bloco 17, São Paulo, SP CEP 05508-000 Brazil; 4 Graduate Program in Endocrinology and Metabology, Universidade Federal de São Paulo, Rua Estado de Israel, nº 639, Vila Clementino, São Paulo, SP CEP 04022-001 Brazil; 5 Nutrition Course, Centro Universitário Estácio de Sá, Rua Erê, 207, Belo Horizonte, MG CEP 30411-052 Brazil; 6 Departament of Obstetrics, Escola Paulista de Medicina, Universidade Federal de São Paulo, R. Napoleão de Barros, 875 - Vila Clementino, São Paulo, SP CEP 04024-002 Brazil; 7 Department of Epidemiology, School of Public Health, Universidade de São Paulo, Av. Dr. Arnaldo, 715 - Cerqueira César, São Paulo, SP CEP 01246-904 Brazil; 8 Department of Preventive Medicine, Escola Paulista de Medicina, Universidade Federal de São Paulo, Campus São Paulo, Rua Botucatu, n° 740, Vila Clementino, São Paulo, SP CEP 04023-062 Brazil

**Keywords:** Gestational diabetes mellitus, Obesity, Gut microbiota, Breastfeeding, Early life events

## Abstract

**Background:**

The incidence of gestational diabetes mellitus (GDM) is increasing worldwide, and has been associated with some changes in the gut microbiota. Studies have shown that the maternal gut microbiota pattern with hyperglycemia can be transmitted to the offspring. The study aimed to evaluate the gut microbiota of obese postpartum women with and without previous GDM and their offspring.

**Methods:**

We evaluated a total of 84 puerperal women who had (n = 40) or not GDM (n = 44), and their infants were also included. Stool samples were obtained 2–6 months after delivery. The molecular profile of the fecal microbiota was obtained by sequencing V4 region of 16S rRNA gene (Illumina^®^ MiSeq).

**Results:**

We found that the gut microbiota structures of the puerperal women and their infants were similar. Stratifying according to the type of delivery, the relative abundance of *Victivallis* genus was higher in women who had natural delivery. Exposure to exclusive breastfeeding was associated with a greater abundance of *Bacteroides* and *Staphylococcus.* The differential abundance test showed correlations to clinical and laboratory parameters. This work showed no difference in the microbiota of obese puerperal women with and without GDM and their offspring. However, breastfeeding contributed to the ecological succession of the intestinal microbiota of the offspring.

**Conclusion:**

This work can contribute to understanding the potential effects of GDM and early life events on the gut microbiome of mothers and their offspring and its possible role in metabolism later in life.

**Supplementary Information:**

The online version contains supplementary material available at 10.1186/s13098-022-00954-2.

## Introduction

Gestational diabetes mellitus (GDM) is the most prevalent complication during pregnancy, and its incidence is increasing worldwide [1]. The presence of GDM has been associated with an increased risk of short (obstetric and neonatal adverse outcomes) and long-term morbidities (type 2 diabetes mellitus, hypertension, and dyslipidemia known as cardiovascular risk factors later in their lives) for both mothers and their offspring [2]. The mechanism by which maternal obesity and diabetes lead to an increased risk of chronic disease in offspring is not well understood. An emerging hypothesis is that these effects may be mediated by the maternal microbiome during pregnancy that is shared with the newborn during pregnancy and delivery.

Microbiota compounds the bacteria communities of the mucosal surface of the respiratory tract, gastrointestinal (GI) tract, urinary tract, and reproductive tract. The GI tract, especially the colon, has the largest microbiota density, defined as “gut microbiota”. Specific gut microbiota changes were identified in women who develop GDM [3–6]. Some studies had shown that gut dysbiosis (an increase in harmful bacteria and a decrease in beneficial bacteria) plays a role in many pregnancy complications, such as preeclampsia, prematurity, and metabolic dysfunction [7–9]. Nevertheless, the role of gut dysbiosis in the pathogenesis of GDM remains unclear, as well as its influence on the intestinal microbiota of the offspring.

There is evidence that the pattern of the mother's gut microbiota with hyperglycemia can be transmitted to the offspring [10–12]. Moreover, the possibility of early modulation of the gut microbiota of these neonatal remains to be understood.

The gut microbiota development in the first years of life is essential for immune function, protection against pathogenic organisms, and several metabolic functions [13, 14]. It is known that neonatal gut microbiota colonization is influenced by gestational conditions, mainly by the microbial community in meconium from neonates’ delivery (C-section or vaginal) mode, as well as perinatal antibiotics and breastfeeding [15]. Considering the role of gut microbiota on the soil of the future of these infants’ s development cardiovascular risk factors [16, 17], the knowledge of the mechanisms of its origin is important [18].

It is known the relationship between obesity and GDM but how the last one can modify the gut microbiota of these mothers and their newborns’ dysbiosis is still in discussion. Also, it is unknown if early life events, such as type of delivery or breastfeeding, could have a role in the intestinal bacterial content of the newborn. In this context, the present study aimed was to evaluate the association of gut microbiota of obese postpartum women with and without previous GDM and early life events with the gut microbiome of their babies.

## Methods

### Design and study population

From September 2018 to December 2019, all pregnant women attending the Normal Gestation Outpatient Clinic of Obstetrics Division and Gestational Diabetes Outpatient Clinic of Diabetes Center of Federal University of São Paulo, SP, Brazil, were invited to participate in the present study. The institutional ethics committee of the Federal University of São Paulo approved the study (Protocol Number: CAAE: 89108618.0.0000.5505), and all participants had signed a consent form [19].

**Fig. 1:**
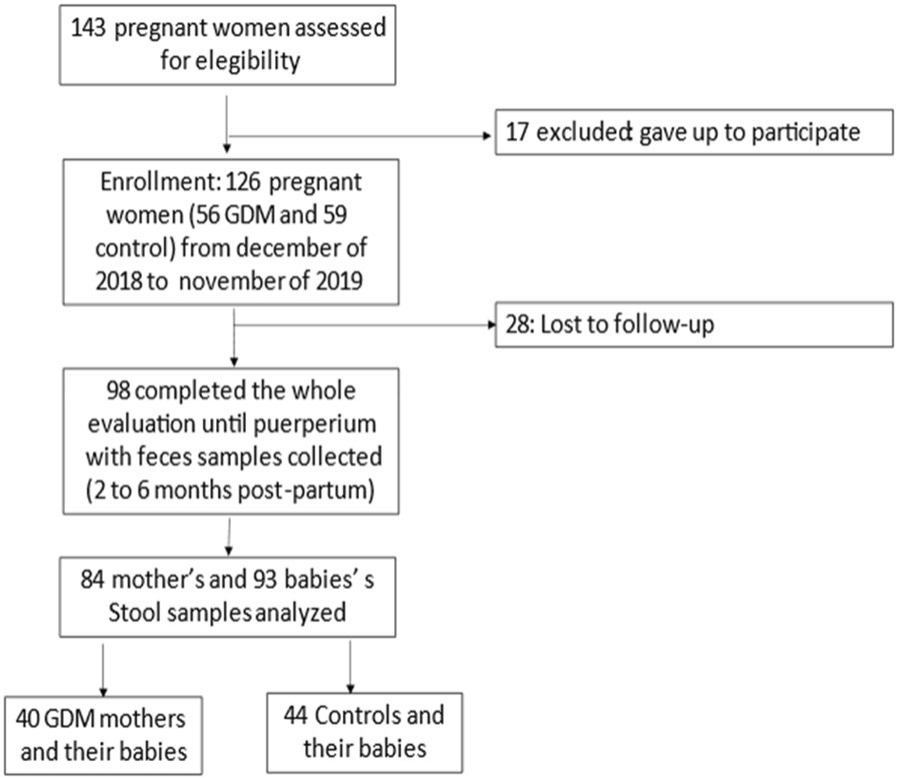
Participant flow chart

Eligibility criteria were age > 18 years, in any trimester of gestation, without known autoimmune disease or chronic use of medications (metformin in particular), or inflammatory bowel disease, and we only included pre-pregnancy overweight or obese women. A total of 143 pregnant women were included in the study, 74 norm glucose-tolerant pregnant controls and 69 GDM women. In the postpartum period (60–180 days after delivery), 50 women with previous GDM and 51 control women with their respective offspring were evaluated. Of these 101 women, 98 collected stool samples for microbiota analysis. We then excluded those women using antibiotics or laxatives or probiotics in the 30 days before stool sample collection. So, forty women with GDM and 44 control women were kept in our analysis. Regarding the offspring, DNA was extracted from 91 stool samples, 46 of which were of controls mothers and 45 of GDM mothers (Fig. 1).

For the diagnosis of GDM, we use the IAPDSG criteria, which is similar to the Brazilian guideline for GDM [20]. However, in this study, GDM was diagnosed in the first trimester when fasting plasma glucose was greater than 100 mg/dL or in the third trimester when at least two points were abnormal during the 75-g oral glucose tolerance (OGTT) (> 92, > 180, > 153 mg/dL at 0, 60 and 120 min respectively) in the third trimester. These criteria were employed to exclude borderline cases of GDM since, in previous studies, the ability to detect differences by comparing the microbiota profiles from normal tolerant and GDM pregnant women with mild hyperglycemia was limited [6].

### Standardized questionnaires

Our study is longitudinal, in which each trimester, the participants were subject to evaluation with standardized questionnaires and anthropometric data collection.

Using standardized questionnaires (Additional file 1: Document 1), puerperium information was obtained under the supervision of trained interviewers. Data collected was the gestational week of delivery, delivery mode, gestational weight gain, maternal–fetal complications, use of medicines or vitamins, and consumption of alcohol or tobacco.

Data from offspring, such as breastfeeding and/or formula use, the introduction of solid foods, and health data, such as hypoglycemia, vaccination, and medications, were collected. Those who received exclusive or predominant (only with eventual tea or water consumption) breastfeeding were considered as “yes” for the breastfeeding variable.

### Anthropometry and blood pressure

Weight and height were obtained on a digital scale (Rice Lake, São Paulo) with 100 g and 0.5 cm, respectively. These measurements were used to calculate BMI. The neck circumference was measured with non-flexible tape (cm) immediately below the cricoid cartilage and perpendicular to the neck's long axis, with the participant seated. The waist circumference was measured with flexible tape (cm) between the last rib and iliac crest in the axillar medium line. The blood pressure (mmHg) was obtained using a mercury sphygmomanometer. Blood pressure was taken three times after a 5-min rest in the sitting position, using a mercury sphygmomanometer adjusted to the brachial circumference. The final systolic and diastolic blood pressure values represent the arithmetic mean of the last two measurements.

### Dietary assessment

All foods and beverages consumed over 3 days, including those consumed outside the home, should be registered in a standardized form. To estimate the size of the portions described more accurately, the nutritionist demonstrated to the patient how to record the information using traditional homemade utensils (cups, cups, cutlery, and plates) and food models. Registration must be on alternate days and cover a weekend day [21]. The total energy value of macro and micronutrients was calculated using Diet Pro software [22], using as reference the Brazilian Food Composition Table (TACO) [23].

### Fecal sample collection and storage

The participants collected fecal samples at home, and the infant samples were taken by collecting the newborn’s diapers with feces in a supplied and standardized container. These samples were brought during a visit to the outpatient Gestational Diabetes Clinics with a maximum of 24-h storage at 2–5 °C. A researcher followed standardized procedures, including antiseptic handling, collection of aliquots in sterile cryotubes, and immediate freezing at − 20 °C. Aliquots were then stored at − 80 °C on the same sampling day until DNA extraction.

### Laboratory tests

Blood samples were collected after overnight fasting, and an oral glucose tolerance test (OGTT) was performed. The samples were immediately centrifuged and analyzed by a certified laboratory. Plasma glucose was determined by the glucose oxidase method. The total plasma cholesterol, HDL-c, and triglycerides were measured by enzymatic colorimetric methods and processed in an automatic analyzer. LDL-c and VLDL-c concentrations will be obtained by difference, using the Friedewald equation. Insulin was measured by the chemiluminescence method.

### Analytic methods

Initially, bacterial DNA from stool samples was extracted using the QIAamp DNA Stool Mini Kit (Qiagen). The amplification of V4 region of the 16S rRNA gene was performed by 25 cycles, using the previously described primers and conditions [24]. Negative controls with a buffer from the DNA extraction kit were included in the PCR runs. The amplicons were pooled and loaded onto Illumina MiSeq clamshell-style cartridge kit v2 at 500 cycles for paired-end 250 sequencing at a final concentration of 12 pM. The library was clustered to a density of approximately 820 k/mm^2^. Sequencing is based on a pool of 100 samples on two GS FLX Picotiter Plate, totaling two races. The raw reads files were processed in the R environment using the dada2 package [25]. During the process, the primers were removed, and the forward and reverse sequences were cut to 180 and 160 bases, respectively. Strings that contained more than two expected errors were removed. The filtered sequences had their errors corrected by the algorithm and were joined to form the ASVs (Amplicon Sequence Variants). The chimeric sequences were also removed, and a sample count table was generated. The taxonomic classification was made using the tag.me package [26] using the model 515F-806R. The beta diversity was calculated using the Jensen-Shannon Divergence matrix on PCoA analysis performed by the ade4 R package [27], and its analysis refers to the variety and complexity of species community. We use the Chao1 wealth estimate and Shannon and Simpson's diversity indexes to calculate the alpha diversity.

### Bioinformatics analysis

Continuous variables were presented as mean (Standard Deviation) and categorical variables as frequency (percentage). Clinical and laboratory variables and maternal–fetal outcomes were compared by Student *t*-test and Mann Whitney test (continuous variables) or Chi-squared test (categorical variables) according to the diagnosis of GDM. Statistical Package for the Social Sciences^®^, v 22.0 (SPSS Incorporation, 2000) was used, and the *p* < 0.05 was considered statistically significant. All data manipulation, analysis, and graphics were performed using R statistical program. PERMANOVA was performed for each site using Adonis function in vegan with Jensen-Shannon distances for differences in the microbiota composition. For each variable, 999 permutations were made. The Wald test of the DESeq2 Package [10.1186/s13059-014-0550-8] and an adjusted p-value filter of *p* < 0.01 were used to identify the differentially abundant genera.

## Results

### Characteristics of study population

#### Mother

Demographic characteristics of 40 GDM cases and 44 non-GDM controls are shown in Table 1. GDM group was older (33.3 ± 5.9 vs. 28.6 ± 6.1 years, *p* = 0.01), and had a higher number of pregnancies (*p* = 0.04) than the control ones. Both groups had similar schooling, family history of diabetes, pre-gestational physical activity practice and BMI, gestational weight gain, tobacco use during gestation and frequencies of natural birth. GDM women had a higher frequency of hypertension than controls; pre-eclampsia, eclampsia and prematurity occurred in the GDM group but not in the control group.

**Table 1:** Pre-gestational, pregnancy, and postpartum data of puerperal women according to the diagnosis of GDM

Pre-gestational and pregnancy data	Controls (n = 44)	GDM (n = 40)	*p*
Age (years)	28.1 (6.1)	33.2 (5.9)	0.01
Caucasian, n (%)	21 (49)	14 (35)	0.18
Schooling, n (%)			0.59
Up to 7 years	1 (2.3)	4 (10.0)	
8–13 years	30 (69.8)	25 (62.5)	
≥ 14 years	12 (27.9)	11 (27.5)	
Number of pregnancies, n (%)			0.04
1 pregnancy	17 (39.5)	8 (20.0)	
2 pregnancies	16 (37.2)	8 (25.0)	
3 or more pregnancies	10 (23.3)	22 (55.0)	
Family history of DM, n (%)	12 (27.9)	16 (41.0)	0.21
Pre-gestational physical activity ≥ 150 min per week, n (%)	11 (255.)	6 (15.0)	0.06
Pre-gestational BMI (kg/m^2^)	29.4 (3.9)	29.8 (3.7)	0.66
Gestational weight gain (kg)	10.4 (6.5)	8.8 (5.0)	0.49
Tobacco use during pregnancy, n (%)	1 (2.4)	2 (5.0)	0.53
Arterial hypertension in pregnancy, n (%)	2 (4.8)	8 (20.0)	0.03
Preeclampsia or eclampsia in pregnancy, n (%)	0	2 (5)	–
Gestational age of delivery (weeks)	39.0 (1.1)	38.3 (1.5)	0.26
Natural birth, n (%)	24 (57.1)	20 (50.0)	0.52
Prematurity < 37 weeks, n (%)	0	3 (0.07)	–
*Postpartum data*			
BMI (kg/m^2^)	29.3 (3.8)	29.5 (3.7)	0.66
Waist circumference (cm)	93.9 (9.1)	92.3 (8.1)	0.53
Systolic BP (mmHg)	112 (10)	116 (9)	0.38
Diastolic BP (mmHg)	73 (8)	75 (6)	0.29
Fasting glycemia (mg/dL)	88.3 (9.0)	94.3 (1.9)	0.16
2 h OGTT glycemia (mg/dL)	98.5 (19.0)	116.3 (38.5)	0.02
Fasting insulinemia (mU/L)	11.5 (5.9)	11.2 (6.4)	0.69
2 h OGTT insulinemia (mU/L)	42.9 (35.2)	53.4 (35.7)	0.46
HbA1c (%)	5.3 (0.3)	5.6 (0.4)	0.02
Total colesterol (mg/dL)	186.1 (30.1)	208.0 (50.6)	0.07
HDL cholesterol (mg/dL)	54.6 (10.9)	56.9 (11.7)	0.75
LDL cholesterol (mg/dL)	110.7 (27.0)	126.6 (45.3)	0.01
Triglycerides (mg/dL)	105.1 (59.3)	112.7 (52.1)	0.75
GamaGT (ng/mL)	23.1 (17.9)	27.4 (22.9)	0.41

Student *t*-test (continuous variables) or chi-square (qualitative variables) were used. Values are mean (SD) or n (%)

*BMI* body mass index, *OGTT* oral glucose tolerance test

Regarding puerperium the anthropometric and blood pressure, glycemia, insulinemia total or HDL cholesterol and triglycerides were no significant difference between these two groups. By the other side, GDM group had higher levels of 2 h glycemia after OGTT [116.3 (38.5) versus 98.5 (19.0) mg/dl, *p* = 0.02], of HbA1c [5.6 (0.4) vs. 5.3 (0.3) %, *p* = 0.02] and of LDL cholesterol [126.6 (45.3) vs. 110.7 (27.0) mg/dl, *p* = 0.01] Table 1.

In comparisons of dietary data between groups, total fiber intake was higher in women who had GDM than the controls [11.9 (9.1–14.5) vs. 6.8 (3.9–13.9) g, *p* = 0.04]. However, intakes of energy total calories/day, and macronutrients (carbohydrates, proteins, and lipids) were similar (Table 2).

**Table 2:** Medians (interquartile ranges) of dietary data of participants according to the presence of GDM diagnosis in their post-partum period

Diet characteristics	Controls (n = 44)	GDM (n = 40)	*p*
Calories (kcals)	1957 (1398–2808)	1284 (1130–1701)	0.40
Carbohydrates (%TEI)	45.9 (38.9–57.3)	49.5 (39.0–57.2)	0.87
Proteins (%TEI)	17.9 (11.3–25.1)	19.8 (12.6–22.9)	0.97
Lipids (%TEI) (g)	32.3 (29.6–37.7)	31.7 (26.9–38.8)	0.42
Saturated fat (%TEI) (g)	6.9 (5.4–11.5)	6.7 (4.9–11.3)	0.84
Monounsaturated fat (%TEI)	7.7 (4.7–9–9)	7.5 (5.3–11.9)	0.42
Polyunsaturated fat (%TEI)	6.9 (5.4–9.9)	7.5 (5.3–10.5)	0.41
Total fiber (g)	6.8 (3.9–13.9)	11.9 (9.1–14.5)	0.04

Mann–Whitney test (continuous variables). Values are median values (IIQ)

*TEI* total energy intake

#### Offspring

A borderline difference indicated that infants of mothers who had GDM were more exclusively breastfed for the first 2–6 months (*p* = 0.058). There was no significant difference between GDM and non-GDM offspring in birth weight or the introduction of food other than the breast milk period. Higher incidences of neonatal complications (as jaundice) in GDM group infants (47.6 vs. 17.0%, p 0.002). (Table 3).

**Table 3:** Offspring data according to the GDM status of the mother

	Controls (n = 48)	GDM (n = 43)	*p*
Birth weight			
Appropiate for gestational age (AGA)	38 (82.6)	30 (69.8)	0.24
Large for gestational age (LGA)	7 (15.2)	9 (20.9)	
Ponderal index	2.82 (0.49)	2.87 (0.28)	0.63
Neonatal complications, n (%)	8 (17.0)	20 (46.5)	< 0.01
Jaundice, n (%)	4 (8.5)	13 (30.2)	< 0.01
Hypoglycemia, n (%)	3 (6.4)	6 (14.0)	0.23
Respiratory distress, n (%)	1 (2.1)	3 (7.0)	0.26
ICU admission, n (%)	2 (4.3)	5 (11.6)	0.19
Malformation, n (%)	1 (2.1)	1 (2.3)	0.95
Exclusive breastfeeding^a^, n (%)	19 (40.4)	26 (60.5)	0.06
Days to introduce foods other than breast milk	80.3 (37.4)	83.1 (44.7)	0.54
Baby weight percentile^a, b^	43.2 (30.2)	40.2 (33.8)	0.58
Baby height percentile^a, b^	37.6 (35.1)	29.6 (31.2)	0.42

*GDM* gestational diabetes mellitus, *ICU* intensive care unit

^a^At the moment of evaluation

^b^Student *t*-test and Mann–Whitney (continuous variables) or chi-square (qualitative variables) were used. Values are mean (SD) or n (%)

### Microbiota composition

#### Mothers

**Fig. 2:**
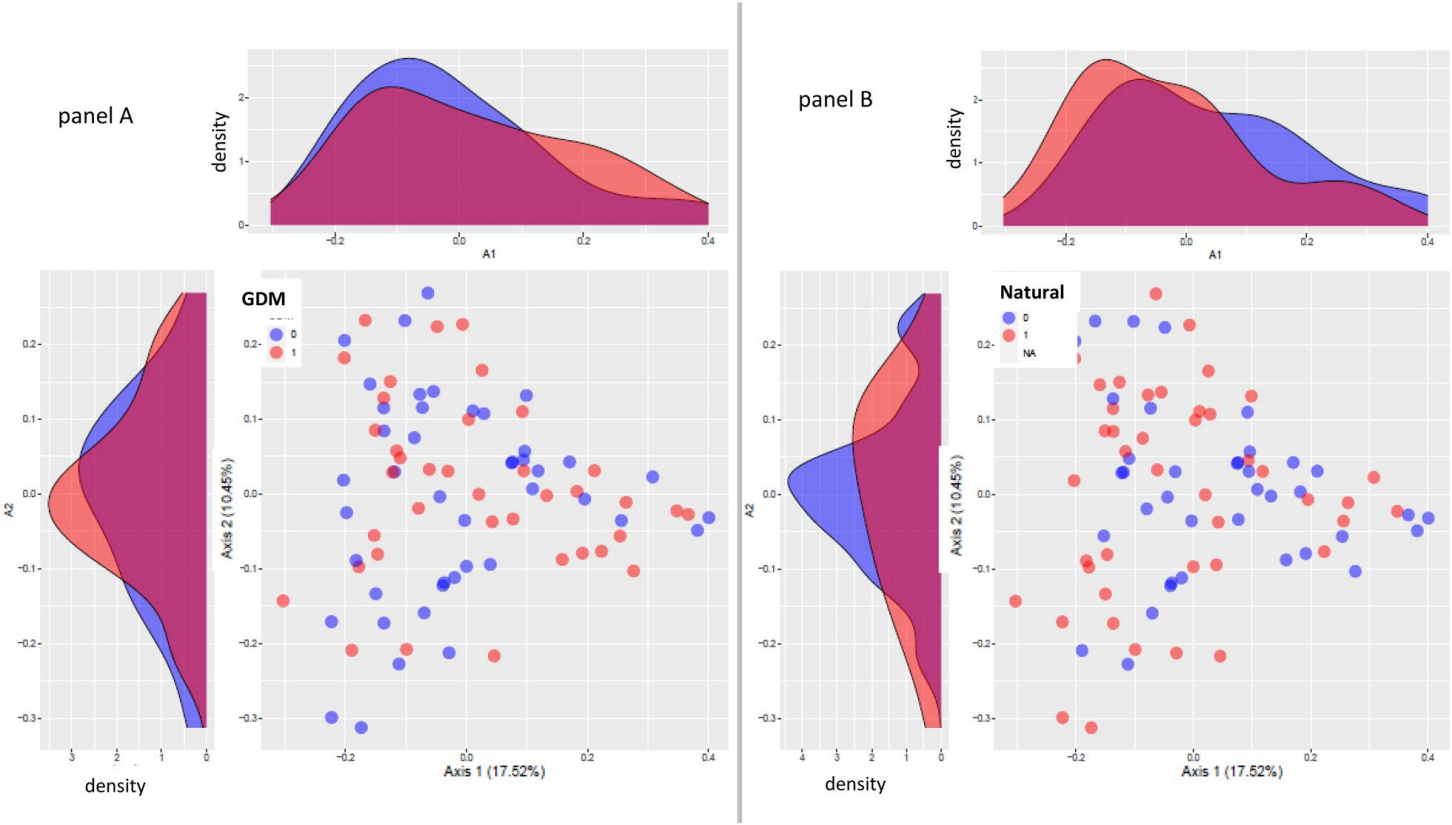
Overall composition of postpartum women’s microbiota with and without GDM (**A**) and according to the type of delivery (**B**) obtained by Principal Coordinate Analysis

**Fig. 3:**
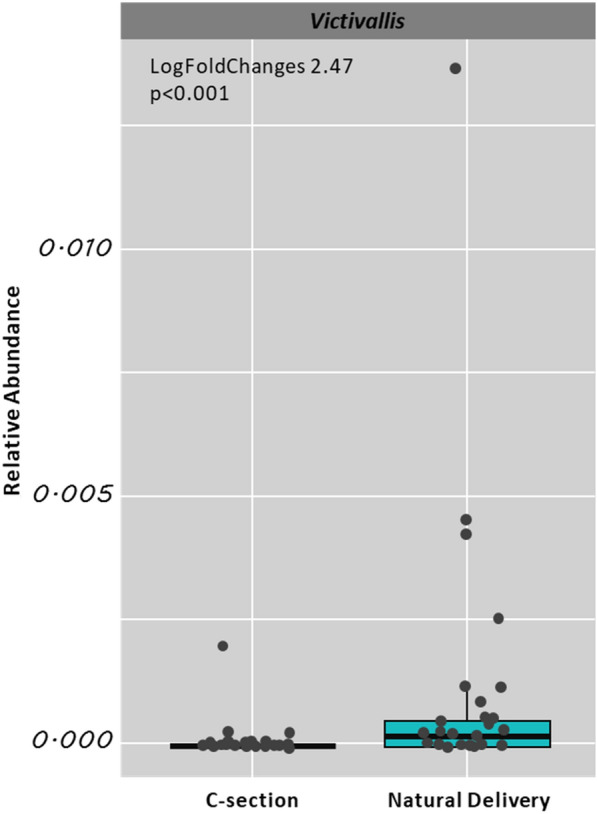
Relative abundance of genus *Victivallis* bacteria according to type of delivery (natural or c section)

The mothers’ gut microbiota composition is shown in Fig. 2. At the time of the postpartum, we did not find a significant difference in the overall composition of the gut microbiota between women who had or not GDM (Fig. 2a), nor when we stratified them according to the type of delivery (Fig. 2b). There was no difference in alpha diversity either (Additional file 1: Fig. S1). Besides a greater relative abundance of *Victivallis* genus (Log-fold change 2.47, *p* = 0.01) in those who had a natural delivery (Fig. 3).

### Correlations between gut microbiota composition and clinical and laboratory data

#### Anthropometrics

We found significant correlations with clinical and laboratory data. Correlations between *Megasphaera* and pre-gestational BMI (rho: 0.31, *p* = 0.005),* Gemella* and waist circumference (rho: − 0.34, *p* = 0.002), *Staphylococcus* and diastolic blood pressure, *Eubacterium hallii group*, and *Phocea* and gestational weight gain (rho: 0.33, *p* = 0.004; rho: 0.34, *p* = 0.002) were detected. (Additional file 3: Table 1S).

#### Nutricional parameters

We also found significant correlations with nutritional data. Correlations between *Lactobacillus* and total calories (rho: 0.49, *p* = 0.004) and *Bifidobacterium* and saturated fat (rho: − 0.48, *p* = 0.004). (Additional file 3: Table 1S).

#### Metabolic parameters

Fasting glycemia was correlated to *Anaerostipes* (rho: 0.31, *p* = 0.005), *Blautia* (rho: 0.31, *p* = 0.005), *Butyrivibrio* (rho: − 0.33, *p* = 0.003) and to *Rikenellaceae.RC9.gut group* (rho: − 0.43, *p* < 0.001). Two-hour plasma glucose was correlated to *Blautia* (rho: 0.31, *p* = 0.008), *Butyrivibrio* (rho: 0.34, *p* = 0.003), *Dorea* (rho: 0.33, *p* = 0.005), *Fusobacterium* (rho: − 0.36, *p* = 0.002), *Lachnospiraceae.FCS020 group* (rho: 0.31, *p* = 0.009), *Lachnospiraceae.NK3A20 group* (rho: − 0.38, *p* = 0.001) and to *Methanosphaera* (rho: − 0.32, *p* = 0.006). Fasting insulinemia correlated with *Succinivibrio* (rho: 0.37, *p* = 0.001). Regarding the lipid profile, triglycerides showed to be correlated to *Dorea* (rho: 0.31, *p* = 0.007) while LDL-c to *Campylobacter* and *Haemophilus* (rho; − 0.33, *p* = 0.004 and rho: 0.30, *p* = 0.007, respectively). (Additional file 3: Table 1S).

#### Offspring

**Fig. 4:**
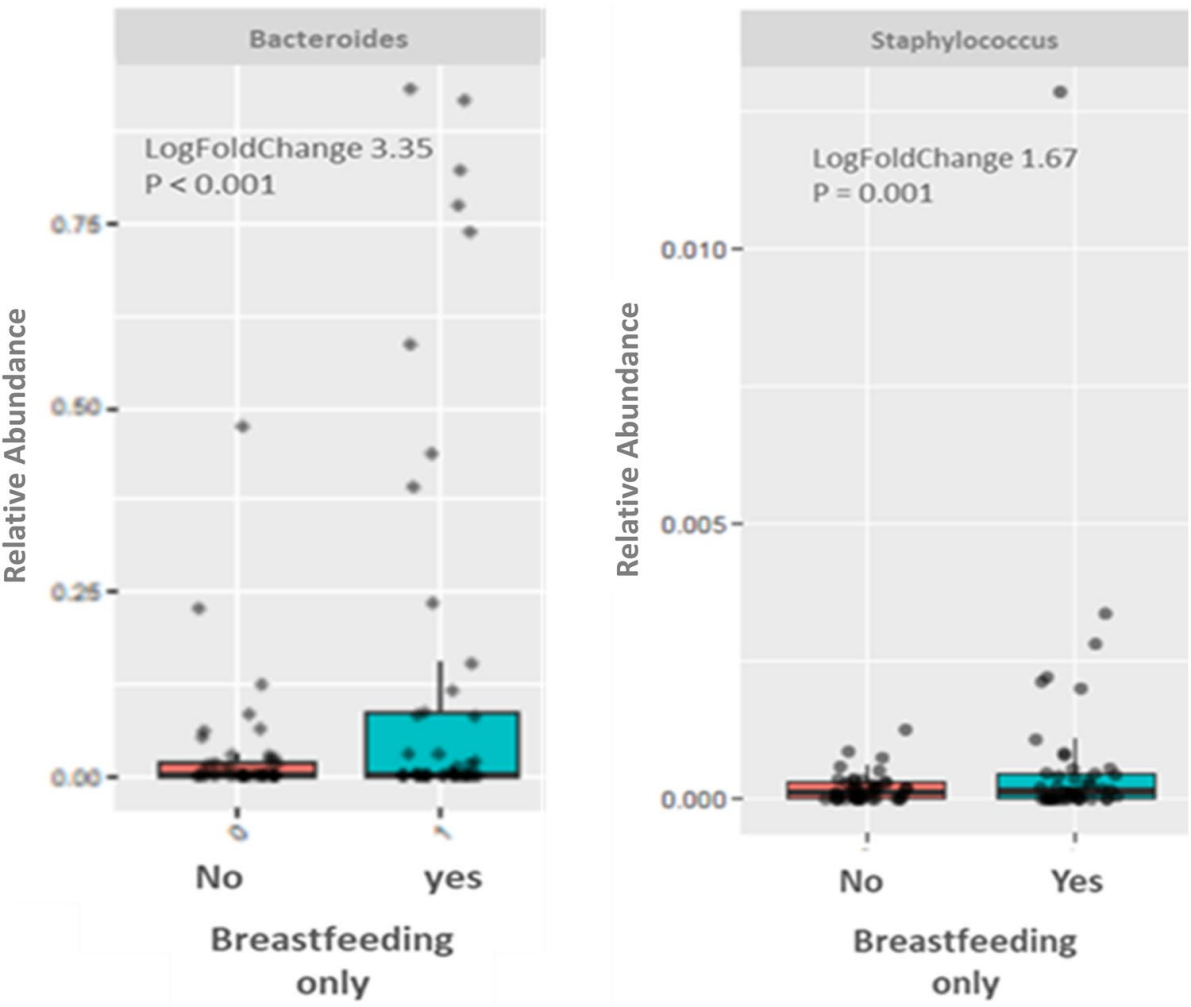
Relative abundance of *Bacteroides* in babies with and without exclusive breastfed for 2 to 6 months

We did not find a difference in gut microbiota from the offspring when they were stratified according to the presence or not of GDM or type of delivery, as we can see by the overlap shown in (Additional file 2: Fig. S2). There was no difference in alpha diversity either (Additional file 2: Fig. S2). In the differential abundance test, offspring exposed to exclusive breastfeeding for 2–6 months showed a greater abundance of *Bacteroides* and *Staphylococcus* (Fig. 4).

The presence of GDM (*p* = 0.47), type of delivery (*p* = 0.13) and exclusive breastfeeding (*p* = 0.71) did not shown interference on the similarity of the gut microbiota composition between mothers and offspring, via beta diversity.

## Discussion

We observed a similar gut microbiota composition of postpartum women stratified by previous or not GDM or type of delivery. Also, we did not detect a difference in the overall composition of the infant’s microbiota according to previous GDM or type of delivery. Considering the relative abundance, the *Victivallis* genus was higher in postpartum women who had a natural delivery. Besides, the microbiota analysis of offspring with exclusive breastfeeding showed a higher relative abundance of genus *Bacteroides* and *Staphylococcus*, which might be compatible with an ecological succession of the intestinal bacterial content.

The women in the GDM group were older, as expected, since advanced age is a risk factor for dysglycemia during pregnancy [28].

The lack of difference when women who had or not GDM and their offspring were compared is similar to results obtained by other investigators. Hasan et al. [29] evaluated 60 women who had GDM and 65 control and their offspring 5 years post-partum and found no significant differences observed in microbiota composition between the two groups and their offspring; mother and her child have a more similar microbiota composition when compared to unrelated children, other mothers, or the children compared to each other.

It is important to mention a limitation of those studies whose analyses were not controlled for body adiposity. It is well known that GDM is accompanied by excess weight that can, per se, modify the microbiota composition [30]. Our study had the advantage of including weight-matched participants in the groups, allowing us to attribute differences found in microbiota profiles to the GDM. Before pregnancy, they were overweight or obese and maintained their nutritional status until the postpartum period. The scientific literature already had shown the relevance of obesity in modulating gut microbiota [30]. A study analyzed the microbiota of infants from overweight and normal-weight mothers and its possible correlation with normal or excessive weight gain during pregnancy [31]. Elevated pre-pregnancy maternal BMI was associated with higher abundances of *Bacteroides*, *Clostridium*, and *Staphylococcus* and lower concentrations of the *Bifidobacterium* group in the infant’s microbiota. After adjusting for pre-pregnancy BMI, *Lactobacillus, Flavonifractor, Erysipelotrichaceae*, and *Gammaproteobacteria* were reduced in neonates from mothers with GDM, reinforcing an independent role of maternal GDM in the infant microbial composition [10]. On the contrary, the actual study did not find differences between GDM and no-GDM groups. The null hypothesis of the actual article could be explained by the homogeneity of the sample regarding the excessive weight in both groups. We hypothesize that being overweight or obese before conception might.

Regarding relative abundance, we detected several correlations of bacterial taxa with clinical, nutritional and laboratory data. Our findings add and corroborate those reported in the literature (Additional file 3: Table 1S). The study by Hasan et al. [29] found that low intestinal *Faecalibacterium/Fusobacterium* ratios corresponded to high blood glucose values in mothers. Genera such as *Prevotella, Streptococcus, Bacteroides*, and Lactobacillus were prevalent in samples from several maternal and neonatal microbiome sites, suggesting GDM response generations' role.

The overall composition of the gut microbiota did not differ according to delivery type, but the relative abundance of *Victivallis* was associated with natural delivery. *Victivallis* is a Gram-negative, coccus-shaped bacteria found in the human digestive tract and is strictly anaerobic. An experimental study in obese rats showed that those with a richer abundance of some genera of bacteria, among them *Victivallis* together with *Barnesiella, Bilophila, Butyricimonas, Clostridium XIVa, Akkermansia, Raoultella,* and *Blautia*, presented a better response to non-drug therapy for weight loss [32]. Furthermore, a randomized clinical study in humans showed that the group that received prebiotic fibers improved the metabolic parameters of glycemia and lipids, together with an increase in bacterial genera such as *Akkermansia, Ruminococcus_2, Victivallis,* and *Comamonas* [33]. Thus, we propose that the microbiome in natural birth may contain composition characteristics associated with potential benefits in metabolism and weight loss.

After the evaluation of microbiota according to the status of having or not GDM, we investigated the role of some early life events in modulating the gut microbiota of the actual sample. We did not detect a difference in the overall composition of the infant’s microbiota according to previous GDM or type of delivery. It is important to consider an age variation among infants, and that intestinal colonization highly depends on exposures in their first 2 years of life [34]. Reports on the microbiota of women who had GDM, and their offspring are controversial. A study compared the 29 GDM offspring to 19 normoglycemic-mother offspring with fecal samples collected during the first week of life [35]. Some *Bacteroides* and *Blautia* oligotypes were shared by the GDM mothers and their offspring, suggesting maternal microbial imprinting. Interestingly, these infants from GDM mothers showed a higher relative abundance of proinflammatory bacteria than infants from healthy women. Another study suggested that GDM could be associated with decreased microbiota richness in the newborns when the meconium DNA from 34 full-term and c-sectioned newborns, in which 20 newborns had mothers diagnosed with GDM, were compared [36]. In another comparison of meconium of 23 newborns from 9 mothers with DM or GDM, and 13 from healthy mothers, the microbiota of the GDM and healthy groups showed lower alpha diversity than that of the DM group [10]. We would like to emphasize that most of these studies also have differences in pre-gestational weigh of the mothers which could be a confounder for the differences that had been found. Our findings evidenced the impact of breastfeeding on the offspring’s microbiota. Babies exclusively breastfed showed a greater abundance of *Bacteroides* and *Staphylococcus*. Since *Bacteroides* is one of the most frequent genera in the stable microbiome, we interpreted that could indicate an ecological progression of intestinal flora. The intestinal microbiome develops until the first 2 years of life, which is considered a window of opportunity for microbial modulation [37]. Similar to our results, *Bacteroides* and *Bifidobacterium* were more abundant in 40-day-aged infants exclusively breastfed than formula-fed ones [38]. *Bacteroides* have been associated with beneficial effects in the earlier neonatal phase. Breast milk factors, like human milk oligosaccharides (HMO), favor *Bacteroides* colonization which is important in activating immunologic functions. This genus and others commensal bacteria stimulate lymphoid elements and enhance intestinal epithelium (microvilli and tight junctions). These also activate the release of mucin by goblet epithelial cells, forming a glycocalyx that breaks down a physical and antibacterial barrier [39]. In an experimental study, germ-free mice colonized with *Bacteroides thetaiodamicron* activated epithelial genes, such as upregulation of polymeric IgA, involved in the barrier function [40]. *Staphylococcus* genus was already described in association with breastfeeding. Comparing fecal microbiota of breast-fed and formula-fed infants, families *Staphylococcaceae* and *Pasteurellaceae* were only found in the breast-fed infant microbiome [41]. The intestine and microbiota maturation undergo a process of colonization primarily by aerobic bacteria and open opportunities for the installation of anaerobes that constitute the main profile of the stable intestinal microbiota in adulthood [42]. We believe that breastfeeding contributes favorably to the process of ecological succession in forming the gut microbiota and contributing to a better immune function.

There is plenty of evidence indicating that feeding type is relevant for early microbial colonization. Breast milk is prebiotic and probiotic in nature, contains HMO and bacteria, and influences infant gut microbiota composition indirectly (transfer of prebiotics) and directly (vertical transmission of bacteria), providing pioneering species. In contrast, formula-fed infants are exposed to different carbohydrates, bacteria, and nutrients, causing different microbial colonization patterns. It has been consistently reported that breastfed infants’ stools compared to formula-feds ones, contain higher levels of *Bifidobacterium* and *Lactobacillus* and lower levels of potential pathogens than those infants with formula-fed [43].

The proposition that gut microbiota acquisition begins intrauterine was contested, and studies have supported that colonization begins at birth [44–46]. Relevant roles in colonization are played by the delivery type, nutrition procedures, and antibiotics use [47–49]. The relevance of the former factors was shown in an analysis of the intestinal microbiome of 120 babies 6 weeks after delivery; the microbiome of cesarean-born babies differed from vaginally delivered ones, but this difference was partially restored by exclusive breastfeeding [50].

Our study has limitations and strengths. As all participants had excess weight, we could not test whether obesity confers an additional impact on the microbiota composition beyond GDM. Infants were very young, so their intestinal flora was not well established. On the other hand, the longitudinal design, long follow-up of women during pregnancy and the puerperium, and early evaluation of their babies, allowed us to think about the hypothesis of ecological succession in forming the gut microbiota.

## Conclusion

We conclude that previous GMD status did not interfere with postpartum mothers' and offspring' overall microbiota composition. However, in the relative abundance, the genus *Victivallis* was high in the natural delivery women. The breastfed infants had a higher relative abundance of *Bacteroides* and *Staphylococcus*. The favorable composition of the gut microbiota in these individuals later in life requires further investigation.

## Supplementary Information

**Additional file 1: Figure S1.** Alpha diversity in postpartum women with (1) or without (0) GDM.

**Additional file 2: Figure S2.** Alpha diversity in babies with (1) or without (0) exposition to GDM.

**Additional file 3: Table S1.** Correlations of bacterial taxa with clinical and laboratory data. **Document S1.** Standardized Questionnaire.

## Data Availability

Data sharing is not applicable to this article as no datasets were generated or analyzed during the current study.
