# Supplementary figures and images for: The gut microbiome of obese postpartum women with and without previous gestational diabetes mellitus and the gut microbiota of their babies
Source: Diabetol Metab Syndr. 2022 Dec 24;14:194. doi: 10.1186/s13098-022-00954-2 (PMC9790115; doi:10.1186/s13098-022-00954-2)

## Slide 1
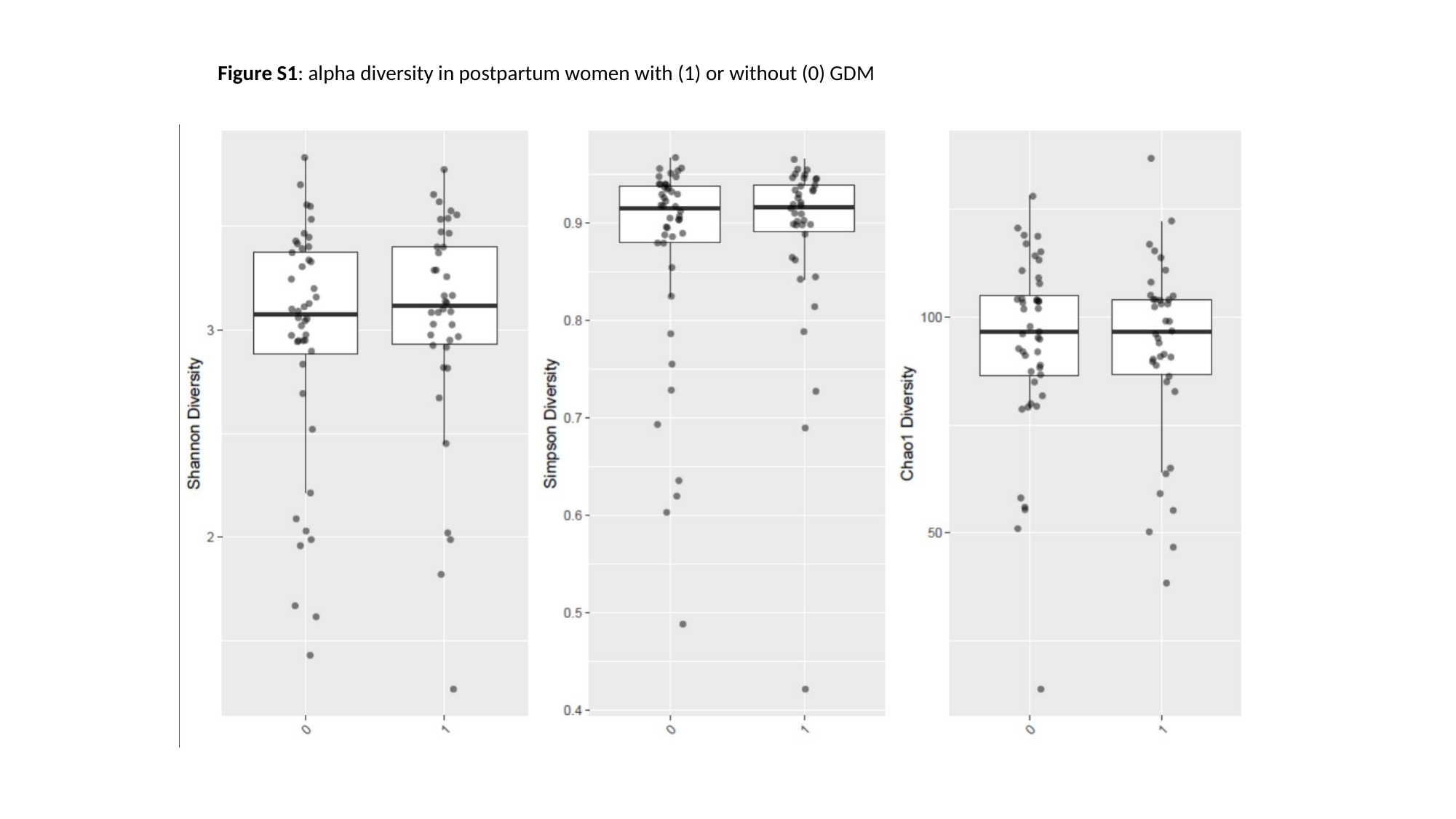

Figure S1: alpha diversity in postpartum women with (1) or without (0) GDM

Supplement: Supplementary file 1 — Additional file 1: Figure S1. Alpha diversity in postpartum women with (1) or without (0) GDM. [file 13098_2022_954_MOESM1_ESM.pptx]

## Slide 1
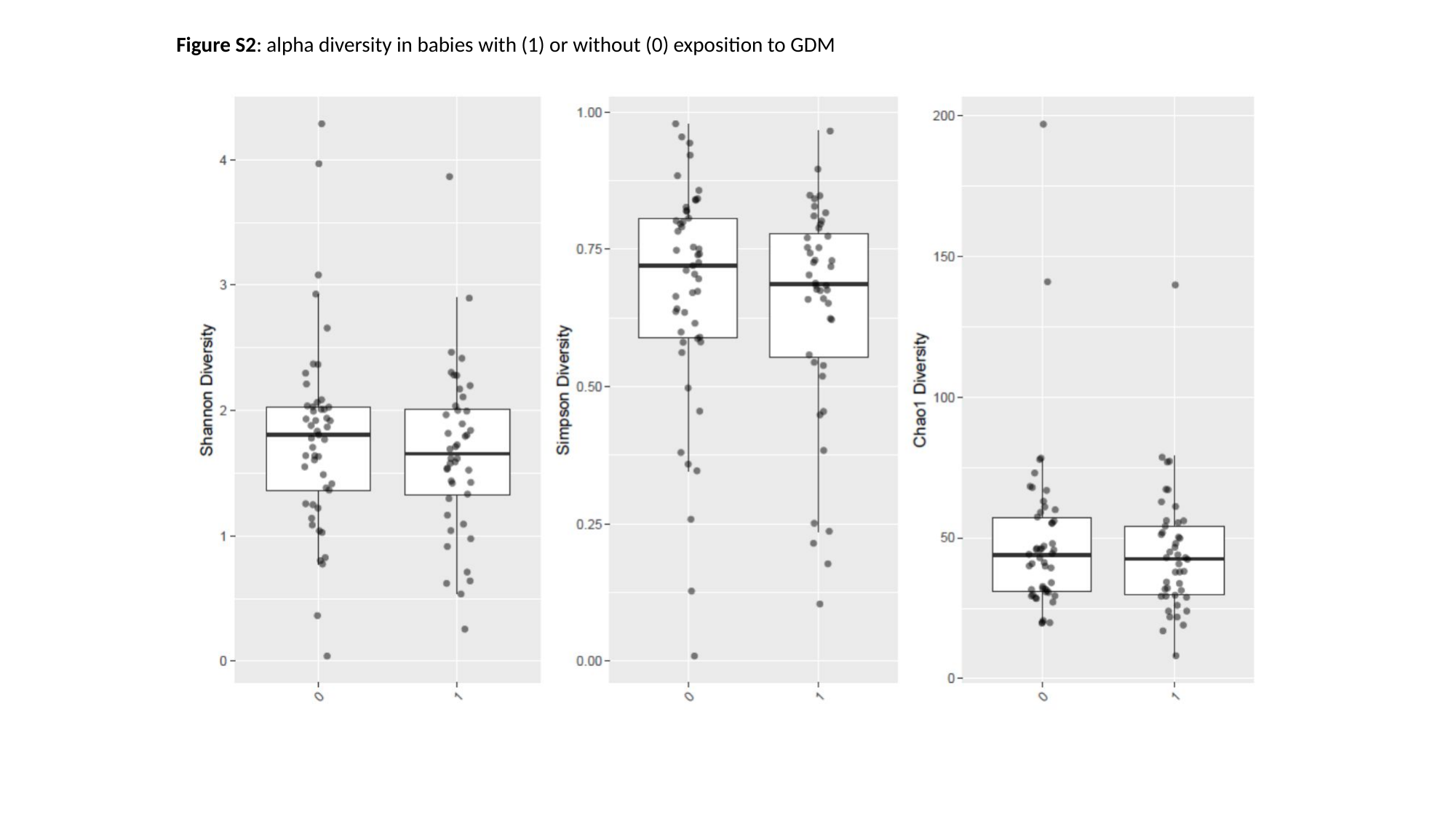

Figure S2: alpha diversity in babies with (1) or without (0) exposition to GDM

Supplement: Supplementary file 2 — Additional file 2: Figure S2. Alpha diversity in babies with (1) or without (0) exposition to GDM. [file 13098_2022_954_MOESM2_ESM.pptx]
