# Supplementary material for: The gut microbiome of obese postpartum women with and without previous gestational diabetes mellitus and the gut microbiota of their babies
Source: Diabetol Metab Syndr. 2022 Dec 24;14:194. doi: 10.1186/s13098-022-00954-2 (PMC9790115; doi:10.1186/s13098-022-00954-2)
Supplement: Supplementary file 3 — Additional file 3: Table S1. Correlations of bacterial taxa with clinical and laboratory data. Document S1. Standardized Questionnaire. [file 13098_2022_954_MOESM3_ESM.docx]

**Table S1: Correlations of bacterial taxa with clinical and laboratory data**

| Bacterias | Maternal variables | rho | p | Phylum |
| --- | --- | --- | --- | --- |
| *Megasphaera* | Pregestational BMI | 0,31 | 0,005 | Firmicutes |
| Gemella | Waist Circumference | -0,34 | 0,002 | Firmicutes |
| Staphylococcus | Systolic BP | -0,33 | 0,004 | Firmicutes |
| *Eubacterium.hallii.group* | Gestational weight gain | 0,33 | 0,004 | Anaerobutyricum |
| *Lactobacillus* | Total calories | 0,49 | 0,004 | Firmicutes |
| *Bifidobacterium* | Saturated fat | -0,48 | 0,004 | Actinobacteria |
| *Phocea* | Gestational weight gain | 0,34 | 0,002 | Firmicutes |
| *Anaerostipes* | Fasting blood glucose | 0,31 | 0,005 | Firmicutes |
|  |  | 0,31 | 0,008 |  |
| *Blautia* | Fasting blood glucose | 0,31 | 0,005 | Firmicutes |
|  | 2-hour OGTT | 0,34 | 0,003 |  |
| *Butyrivibrio* | Fasting blood glucose | -0,33 | 0,003 | Firmicutes |
| *Rikenellaceae.RC9.gut.group* | Fasting blood glucose | -0,43 | 0,000 | Bacteroidetes |
| *Dorea* | 2-hour OGTT | 0,33 | 0,005 | Firmicutes |
| *Dorea* | Triglycerides | 0,31 | 0,007 |  |
| *Fusobacterium* | 2-hour OGTT | -0,36 | 0,002 | Fusobacteriota |
| Lachnospiraceae.FCS020.group | 2-hour OGTT | 0,31 | 0,009 | Firmicutes |
| *Lachnospiraceae.NK3A20.group* | 2-hour OGTT | -0,38 | 0,001 |  |
| *Methanosphaera* | 2-hour OGTT | -0,32 | 0,006 | Euryarchaeota |
| *Succinivibrio* | Fasting Insulinemia | 0,37 | 0,001 | Gammaproteobacteria |
| *Campylobacter* | LDL colestherol | -0,33 | 0,004 | Proteobacteria |
| Haemophilus | LDL colestherol | 0,30 | 0,007 | Proteobacteria |

**BMI, body mass index. BP, blood pressure. OGTT, oral glucose tolerance test**

**Document Ss1 – Standardized Questionnaire**

**Postpartum**

| Exam: | | |
| --- | --- | --- |
| Height: | Weight: | Delivery date |
| Neck Circumference: | Waist Circumference: | Blood Pressure: |

- Do you currently use:

( ) Alcohol. How much per day? _________ Per week? _________

( ) Smoking. How many cigarettes a day?? ___________

( ) Drugs.

( ) Vitamins. Which? __________________________________________________

- Have you changed your diet a lot after giving birth?

( ) Yes How? ______________________________________________________

( ) No

( ) I do not know

- What type of delivery was your baby born?

( ) Natural

( ) Cesarean

( ) Forcips

( ) I do not know

- How many weeks was your baby born?

___________________

( ) I do not know

- What was your baby's birth weight? ________________g
- Did your baby have any health problems at birth?

( ) No.

( ) Yes. Which one(s)?

( ) Jaundice

( ) Hypoglycemia

( ) Respiratory distress

( ) ICU admission

( ) Infecction

( ) Others: _______________________________________

( ) I do not know

- Are you breastfeeding (exclusive or not)?

( ) Yes

( ) Exclusively breastfed for ____months

( ) Formula started at ____months of baby's life.

What is the formula name?________________________________

( ) No

- Has your baby fed anything other than milk until now?

( ) No

( ) Yes Which one? ______________________________________________________

( ) I do not know

- Have you had any drug treatment while breastfeeding?

( ) Asthma

( ) Contraceptive

( ) Antibiotics

( ) Others: ________________________________

( ) No

( ) I do not know

- Has your baby had any health problems so far?

( ) Yes Which one? __________________________________________________

( ) No

( ) I do not know

- Has your baby had the recommended vaccines for his age?

( ) Yes. Which one(s)? _____________________________________________________

( ) No. Which one(s)? ____________________________________________________

( ) I do not know

- Has your baby taken any medication other than vitamins to date?

( ) Yes Which one? _______________________________________________________

( ) No

- Do you have a pet at home?

( ) Yes Which one? _______________________________________________________

( ) No
